# Causal Relationships between Air Pollutant Exposure and Bone Mineral Density and the Risk of Bone Fractures: Evidence from a Two-Stage Mendelian Randomization Analysis

**DOI:** 10.3390/toxics12010027

**Published:** 2023-12-30

**Authors:** Xiao Hu, Yan Zhao, Tian He, Zhao-Xing Gao, Peng Zhang, Yang Fang, Man Ge, Yi-Qing Xu, Hai-Feng Pan, Peng Wang

**Affiliations:** 1 Teaching Center for Preventive Medicine, School of Public Health, Anhui Medical University, 81 Meishan Road, Hefei 230032, China; 2245010561@stu.ahmu.edu.cn; 2 Institute of Kidney Disease, Inflammation & Immunity Mediated Diseases, The Second Hospital of Anhui Medical University, Hefei 230032, China; zyzbj1999@163.com (Y.Z.); 2245010384@stu.ahmu.edu.cn (T.H.); gzx19980917@163.com (Z.-X.G.); 18355318896@163.com (P.Z.); yangf_ahmu@sina.com (Y.F.); gemanahmu2022@163.com (M.G.); xuyiqing7636@163.com (Y.-Q.X.); 3 Department of Epidemiology and Biostatistics, School of Public Health, Anhui Medical University, 81 Meishan Road, Hefei 230032, China

**Keywords:** air pollution, bone mineral density, osteoporosis, bone fractures

## Abstract

A number of studies from the literature have suggested that exposure to air pollutants is associated with a declined bone mineral density (BMD), and increased risks of osteoporosis (OP) and bone fractures. This study was performed to systemically assess the genetically causal associations of air pollutants with site-/age-specific BMD and risk of bone fractures with the implementation of two-sample Mendelian randomization (TSMR) and multivariate Mendelian randomization (MVMR). The TSMR analysis was implemented to infer the causal associations between air pollutants and BMD and the risk of bone fractures, additional MVMR analysis was used to further estimate the direct causal effects between air pollutants and BMD, the occurrence of OP, and bone fractures. The results showed that NOx exposure contributed to lower femoral neck BMD (FN-BMD) (β = −0.71, 95%CI: −1.22, −0.20, *p* = 0.006) and total body BMD (TB-BMD) (β = −0.55, 95%CI: −0.90, −0.21, *p* = 0.002). Additionally, exposure to PM10 was found to be associated with a decreased TB-BMD (B β = −0.42, 95%CI: −0.66, −0.18, *p* = 0.001), further age-specific subgroup analysis demonstrated the causal effect of PM10 exposure on the decreased TB-BMD in a subgroup aged 45 to 60 years (β = −0.70, 95%CI: −1.12, −0.29, *p* = 0.001). Moreover, the findings of the MVMR analysis implied that there was a direct causal effect between PM10 exposure and the decreased TB-BMD (45 < age < 60), after adjusting for PM2.5 and PM2.5 —10 exposure. Our study provides additional evidence to support the causal associations of higher concentrations of air pollutant exposure with decreased BMD, especially in those populations aged between 45 to 60 years, suggesting that early intervention measures and public policy should be considered to improve public health awareness and promote bone health.

## 1. Introduction

Osteoporosis (OP) is a chronic metabolic disease characterized by reduced bone mass, microarchitectural deterioration, and an increased risk of fragility fractures [1]. It poses a significant public health challenge, with approximately 200 million people suffering from OP each year [2]. Bone mineral density (BMD) measurements are recommended as the most reliable tool for the diagnosis of OP and assessment of bone health [3]. Age and gender-specific BMD measurements have suggested that both the BMD and bone mass gradually decreased in the body with ageing process, and showed a gender specificity during BMD decline trend [4]. As a multifactorial disease, the exact etiology of OP is still not well understood, it has been revealed that genetic susceptibility, ageing, lifestyle, and medical conditions, etc., contribute to the onset and development of OP [5]. In recent years, emerging evidence has suggested that environmental factors may play an important role in the pathogenesis of OP [6].

Air pollutants, as an important component of these environmental factors, are defined as harmful concentrations of gaseous substances, particulate matter, and volatile substances [7]. A large number of studies from the literature have demonstrated that short- or long-term exposure to air pollutants could cause chronic inflammation, induce the disturbance of oxidative stress and DNA damage, result in serious negative effects on human health, and lead to a series of disorders that involve the respiratory, cardiovascular, and central nervous systems [8,9]. An earlier cohort study revealed that exposure to air pollutants exhibited a harmful effect on bone health, where high levels of air pollutant exposure were strongly associated with reduced BMD, and increased risk of late-life bone fractures [10]. A similar finding was also observed in a population-based retrospective cohort study, in which exposure to air pollutants increased the risk of occurrence of OP from 39% to 89% in Taiwanese residents [11]. Given the fact that several previous findings were based on observational studies, there remain, however, numerous unmeasured confounding factors and potential biases that might affect the validity and reliability of the observed associations, and the causal links between air pollution and BMD/bone fractures remain obscure.

Mendelian randomization (MR) is a cutting-edge statistical approach that leverages genetic variants as instrumental variables (IVs) to draw conclusions about causality between exposure and outcome. MR offers superior control for confounding factors and reverse causal associations compared to traditional observational studies, thus providing valuable genetic evidence for disease prevention and treatment [12]. This innovative method holds promise for advancing our understanding of complex diseases and informing public health policies.

In the present study, we aimed to infer the causal associations of air pollution exposures with site-/age-specific BMD and the risk of bone fractures via the implementation of two-sample MR (TSMR) and multivariate MR (MVMR) analyses, in order to identify and understand the causal roles of air pollution involved in the development of OP and bone fractures, which would be beneficial for the improvement of prevention measures and the overall quality of life in these populations.

## 2. Methods

### 2.1. Study Design

In MR analysis, single-nucleotide polymorphisms (SNPs) are commonly selected as IVs for estimates of exposure–outcome causal associations, but they must satisfy three key assumptions [13]. First, the correlation assumption requires that IVs must be highly correlated with exposure factors in order to avoid the possible bias of weak IVs [14]. Second, the exclusion restriction hypothesis must ensure that outcomes are solely influenced by exposure and not by any other factors, that means there is no potential for multiple causal pathways [15]. Third, the independence assumption requires that IVs should be free of confounding factors in the exposure–outcome association [16].

**Figure 1:**
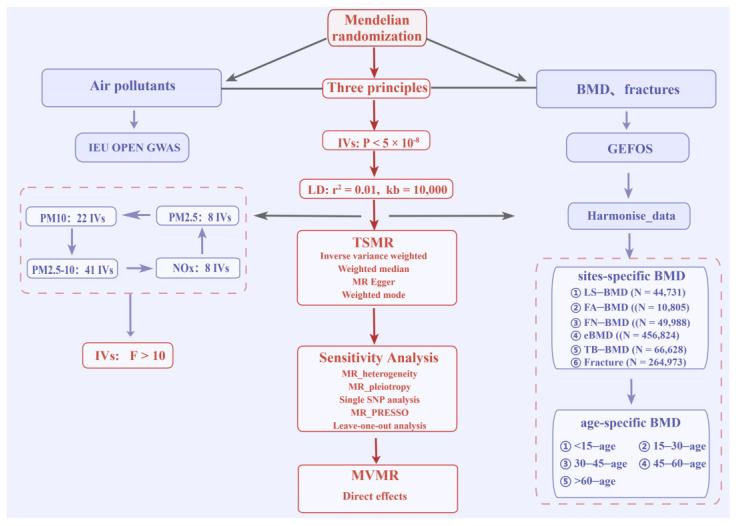
The study design of causal inference between air pollutants and BMD/bone fracture risk. PM: particulate matter; NO_X_: nitrogen oxides; BMD: bone mineral density; FA: forearm; FN: femoral neck; LS: lumbar spine; eBMD: estimated heel BMD; TB: total body BMD; GEFO: Genetic Factors for Osteoporosis Consortium.

The current study was a two-stage MR design (Figure 1). Initially, the causal relationships of air pollutants with site-/age-specific BMD and the risk of bone fractures were evaluated using the univariate MR analysis, where the exposure phenotype for air pollutants included particulate matter 2.5 (fine particulate matter less than 2.5 microns in diameter, PM2.5), particulate matter 2.5–10 (fine particulate matter between 2.5 and 10 microns in diameter, PM2.5–10), particulate matter 10 (fine particulate matter less than 10 microns in diameter, PM10), and nitrogen oxides (NOx). Given the observed causal associations between air pollutants and site- or age-specific BMD and the risk of bone fractures, further MVMR analysis was performed to explore the presence of the direct causal effects of single air pollutants, after adjusting for the confounding effect of the other air pollutants.

### 2.2. Exposure Data Sources and IV Selection

Genetic instruments for air pollutants were obtained from the IEU OPEN GWAS database (https://gwas.mrcieu.ac.uk/, accessed on 15 May 2023), and we used IVs to explore the association between air pollutants and BMD, as well as fractures. The exposure datasets for three types of particulate matter (PM2.5, PM2.5–10, and PM10) were obtained from the UK Biobank, which compiled 423,796 participants of European ancestry. The concentrations of PM2.5, PM10, and PM2.5–10 were estimated with the use of land use regression (LUR) models, developed by the European Study of Cohorts for Air Pollution Effects (ESCAPE) project, at the home addresses of the participants [17]. In addition, NOx data was obtained from the MRC-IEU database, which was exported from the GWAS pipeline using UK Biobank’s Pheasant-derived variables.

Based on the three core assumptions of the MR analysis (correlation, independence, and exclusivity), we conditioned the selection of IVs to ensure the validity of causal estimates [18]. To be first, we only chose IVs from individuals of European ancestry to reduce potential bias from population stratification [19]. Subsequently, in accordance with the assumption of correlation, based on the threshold of *p* < 5 × 10^−8^ and the linkage disequilibrium (LD) (r^2^ = 0.01, kb = 10,000), quantities of 8, 0, 22, and 8 genome-wide associated SNPs were selected for PM2.5, PM2.5–10, PM10, and NOx, respectively. However, as there were no available SNPs selected for PM2.5–10 at the level of *p* < 5 × 10^−8^, we relaxed the threshold to *p* < 1 × 10^−5^ for IV selection and found 41 PM2.5–10-associated SNPs. Due to the lack of available proxies in some of the exposure–outcome analyses, we excluded some SNPs as they were not present in the outcome. In addition, the correlation strengths of enrolled IVs were assessed using the *F* statistic, with the equation: F = (R^2^ × (n − k − 1))/(k × (1 − R^2^)); R^2^ = 2 × ((1 − MAF) × MAF × beta) [20], and the results showed that the *F* value of each selected IVs was greater than 10, indicating that the selected IVs were not prone to the influence of weak IVs. To ensure the independence of IVs, sensitivity analysis was performed to assess the impact of residual confounding of the results, and multivariate MR (MVMR) analysis was also implemented to investigate the direct causal effects after controlling for potential confounders. Furthermore, to meet the exclusion restriction hypothesis, the MR pleiotropy residual sum and outlier (MR-PRESSO) and MR-Egger methods were employed to detect the horizontal pleiotropy. Finally, there were a total of 79 air pollutant-associated SNPs, including 8 PM2.5-associated SNPs, 41 PM2.5–10-associated SNPs, 22 PM10-associated SNPs, and 8 NOx-associated SNPs.

### 2.3. Outcome Data Sources

For the outcome datasets, we used BMD as the outcome to represent the phenotype of OP, due to the varied BMD among the different body sites and age subgroups, the genetic summary data regarding five site-specific BMD measurements [lumbar spine BMD (LS-BMD), forearm BMD (FA-BMD), femoral neck BMD (FN-BMD), estimated from quantitative heel ultrasounds BMD (eBMD), and total body BMD (TB-BMD)] and five age-specific BMD measurements (age ≤ 15, 15 < age ≤ 30, 30 < age ≤ 45, 45 < age < 60 and age ≥ 60) were derived from the three large GWAS analysis consortiums based on reports by Zheng et al., Kemp et al., and Medina-Gomez et al., respectively [13,21,22], where the FN-BMD, LS-BMD, FA-BMD, TB-BMD, and five age-specific BMD measurements were measured by dual-energy X-ray absorptiometry (DEXA), and the eBMD was detected by quantitative ultrasonography. Considering the close relationships of OP and fractures, summary statistics for bone fractures were obtained from a publicly available GWAS by Morris et al. [23]. All of the genetic summary-level data were downloaded from Genetic Factors for Osteoporosis Consortium (GEFOS), no additional ethical checks were therefore required.

### 2.4. Participant Overlap Assessment

During the causal inference of MR analysis, there has often been IV bias due to a high overlap of the samples, which would create the possibility of a type 1 error [24]. The reliability and validity of MR analysis could be accepted with no or minor overlap between exposures and outcomes (sample overlap < 10%) [25]. The sample overlap between the datasets of air pollutants and OP/bone fractures was calculated in using an online tool (Bias and Type 1 error rate for Mendelian randomization with sample overlap [shinyapps.io]).

### 2.5. Statistical Analysis

#### TSMR Analysis

Initially, the TSMR analysis was conducted to estimate the causal effects of four kinds of air pollutants (including PM2.5, PM10, PM2.5–10, and NOx) with site-/age-specific BMD and risk of bone fractures. The strength of genetic instruments for air pollutant correlations were quantified using the F-statistic, and the efficacy of the IVs was further assessed for all SNPs; statistical power was computed with the implementation of the online tool “mRnd” (https://shiny.cnsgenomics.com/mRnd/, accessed on 17 May 2023). The inverse variance weighted (IVW) and weighted median (WM) models were defined as the main analytical methods for the judgement of causal inference, and an additional MR-Egger model and weighted mode were also conducted. Among these four types of analytical methods, the IVW method presented as a reliable tool that could provide the precise causal effects between exposure and outcome, especially in the absence of horizontal pleiotropy [26]. In addition, considering the potential bias in the case of pleiotropy when performing IVW method, the WM method was constructed to verify the accuracy and stability of the results. When there were more than 50% invalid IVs present, the WM method yielded the most accurate results; it not only reduces the occurrence of Type I errors, but also provides a high degree of accuracy in assessing causal associations [27]. Considering the multiple tests during the causal inference between air pollutants and BMD/bone fractures, the false discovery rate (FDR) correction was implemented to adjust the *p*-values of the tests, in order to minimize the number of false positives [28]. Both the IVW and WM results with *p* < 0.05 and FDR *q*-value < 0.05 were defined as the presence of strong evidence of causality.

### 2.6. MVMR Analysis

MVMR is an extension of univariate MR that uses genetic variants associated with multiple potential exposures to estimate the direct effect of any single exposure on the outcome [29]. Considering that there might be a potential confounding effect between different air pollutants during the causal inference, the MVMR analysis was constructed to assess the overall causal effect of air pollutants on BMD and the risk of bone fractures, as well as to evaluate the independently causal link between single air pollutants and BMD and risk of bone fractures after controlling for the influence of other air pollutants (independence assumption) [30]. A *p*-value < 0.05 in MVMR analysis was denoted to be statistical significance.

#### Sensitivity Analysis

To ensure the stability and reliability of our results, we conducted sensitivity analyses using several methods. First, to investigate possible horizontal pleiotropy, the MR-PRESSO method was employed to detect and correct for horizontal pleiotropy [15], and it was found that there was no significant horizontal pleiotropy with a *p*-value greater than 0.05, indicating that IVs did not affect the outcome through pathways independent of exposure [31]. Second, Cochran’s *Q* statistic was calculated to assess the degree of heterogeneity among the included IVs, where a *p*-value > 0.05 indicated no marked heterogeneity among the IVs, suggesting that the causal associations were not influenced by the individual SNP effects [31]. In addition, the leave-one-out (LOO) method was used to further evaluate the robustness of causality by excluding single SNPs at the time of the analysis and reassessing the impact on the overall causal estimates [32].

All statistical analyses were conducted using R version 4.2.2 software with the implementation of “TwoSampleMR”, “MRPRESSO”, and “MendelianRandomization” packages. All results were visualized in the forms of scatter plots, forest plots, funnel plots, and leave-one-out plots, with the use of the “ggplot2” and “forestplot” packages.

## 3. Results

### 3.1. Baseline Characteristics

Given the aforementioned criteria of IV selection, 79 air pollutant-associated SNPs were screened for causal estimates in the present study, which comprised 8 PM2.5-associated SNPs, 41 PM2.5–10-associated SNPs, 22 PM10-associated SNPs, and 8 NOx-associated SNPs, respectively (Supplementary Table S1). The *F*-statistics for PM2.5, PM2.5–10, PM10, and NOx were 34.526, 36.534, 21.925, and 35.466, respectively, suggesting that the selected IVs were sufficiently robust, and were not prone to the influence of weak IVs. Moreover, the calculation of participant overlaps found a lower sample overlap between the datasets of air pollutants and OP/bone fractures (Supplementary Figure S1), indicating that the causal estimates of the present study were less likely to be affected by Winner’s curse bias.

### 3.2. Stage 1 Causal Associations between Air Pollutants and BMD and Bone Fracture Risk

**Figure 2:**
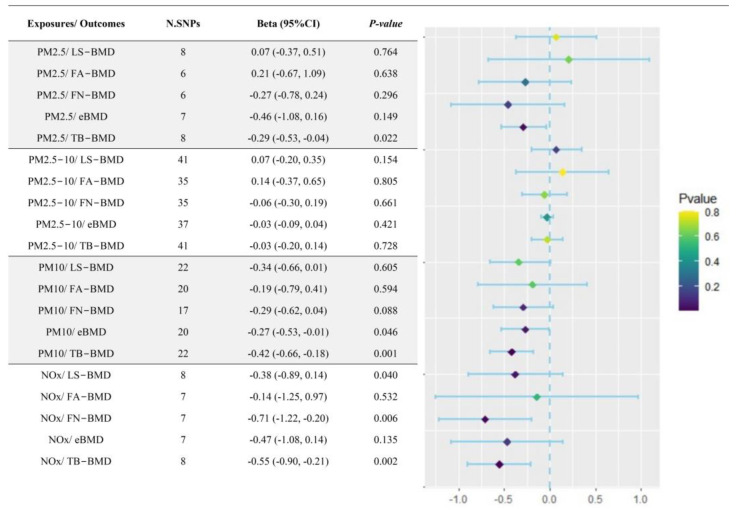
Forest plot of the causal effects of air pollutants on site-specific BMD. PM: particulate matter; NOx: nitrogen oxides; BMD: bone mineral density; FA: forearm; FN: femoral neck; LS: lumbar spine; eBMD: estimated heel BMD; TB: total body BMD; N.SNPs: number of single-nucleotide polymorphisms.

Initially, the causal associations between four types of air pollutants (PM2.5, PM2.5–10, PM10, and NOx) and five site-specific BMD measurements (LS-BMD, FA-BMD, FN-BMD, eBMD, and TB-BMD) and the risk of bone fractures were explored by conducting univariate TSMR analysis. The results of the IVW method together with the FDR correction revealed that NOx exposures were causally linked with the lower FN-BMD (β = −0.71, 95%CI: −1.22, −0.20, *p* = 0.006) and TB-BMD (β = −0.55, 95%CI: −0.90, −0.21, *p* = 0.002), and PM10 exposures were causally associated with the decreased TB-BMD (β = −0.42, 95%CI: −0.66, −0.18, *p* = 0.001) (Figure 2 and Supplementary Figure S2). The above findings were also supported by the WM method (Supplementary Table S2). In addition, we did not detect the presence of horizontal pleiotropy and marker heterogeneity (both *p* > 0.05) (Supplementary Table S2), and further sensitivity analysis with LOO method revealed that no single SNP drove these results after stepwise elimination of individual SNPs (Supplementary Figures S3 and S4).

**Figure 3:**
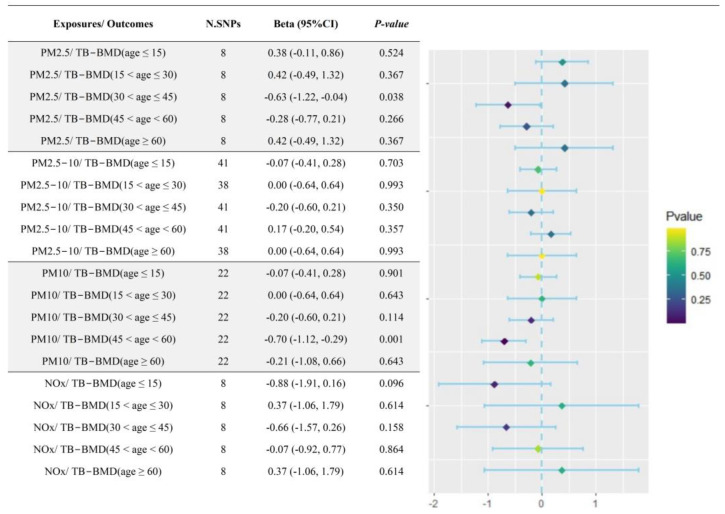
Forest plot of causal effects of air pollutants on age−specific BMD. PM: particulate matter; NOx: nitrogen oxides; BMD: bone mineral density; N.SNPs: number of single-nucleotide polymorphisms.

In order to further investigate the causal effects of air pollutants on age-specific BMD, five subgroups of age-specific TB-BMD (including age ≤ 15, 15 < age ≤ 30, 30 < age ≤ 45, 45 < age < 60 and age ≥ 60) were applied to represent as the phenotype outcomes for causal inference. The results of IVW method and FDR correction observed a negatively causal association of PM10 exposure with a decreased TB-BMD in aged 45 to 60 years group (β = −0.70, 95%CI: −1.12, −0.29, *p* = 0.001) (Figure 3 and Supplementary Figure S5), further WM method supported the robustness of the IVW results (Supplementary Table S3).

**Figure 4:**
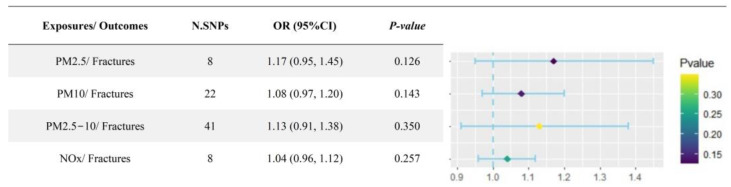
Forest plot of the causal associations between air pollutants and an increased risk of bone fractures. PM: particulate matter; NOx: nitrogen oxides; N.SNPs: number of single-nucleotide polymorphisms; OR: odds ratio.

As previously described, OP patients were at higher risk of the occurrence of bone fractures; therefore, the additional TSMR analysis was performed to infer the causality between air pollutants and the risk of bone fractures, and the results indicated that there were no obviously causal effects of air pollutant exposure on the risk of bone fractures (Figure 4 and Supplementary Table S4).

### 3.3. Stage 2 Direct Causal Effects of Single Air Pollutants on Age-Specific TB-BMD

**Figure 5:**
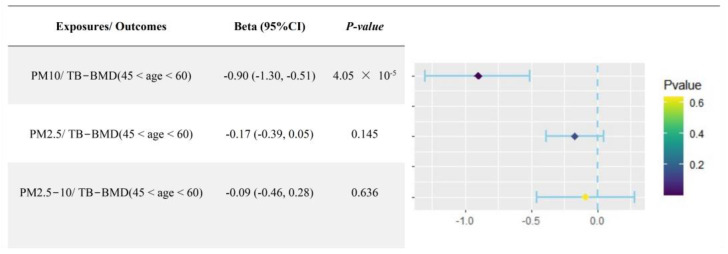
Direct effects of PM10 exposure on age-specific TB−BMD (45 to 60 years) after adjusting for PM2.5 and PM2.5–10. PM: particulate matter; BMD: bone mineral density.

Considering the possibility of interactional effects of multiple particulate matters on BMD simultaneously, the MVMR analysis was implemented to evaluate the direct effects of PM10 on age-specific TB-BMD (45 < age < 60) after correcting for the influences of PM2.5 and PM2.5–10. The results of the MVMR analysis found that single exposure to PM10 had a directly causal effect on decreased TB-BMD in the subgroup aged between 45 to 60 years (β = −0.91, 95%CI: −1.30, −0.51, *p* = 4.05 × 10^−5^) (Figure 5), suggesting that there was an independent effect of PM10 exposure on bone damage in the vulnerable population aged between 45 to 60 years.

## 4. Discussion

Over the past two decades, a large number of studies from the literature have demonstrated that environmental pollution exerted detrimental effects on various aspects of human health. Air pollution is one of the most significant contributors to overall environmental pollution. It occurs when harmful gases, particulate matter, and chemicals are released into the air. Long-term exposure to air pollution has also been linked to an increased risk of heart disease, stroke, lung cancer, and premature death [33]. Carla et al. highlighted the connection between air pollutant exposure and excessive body fat, and this finding was further supported by animal experiments [34]. It is well-known that the health risks associated with air pollution could affect people of all age groups, with particularly severe consequences for vulnerable populations, such as the elderly [35].

Previous studies have shown that long-term exposure to air pollution can have negative effects on bone health. Specifically, it has been found that exposure to particulate matter and nitrogen dioxide (NO_2_), two common components of air pollutants, can lead to reduced BMD and increased risk of bone fractures in later life [36]. Prada et al. firstly explored the effects of air pollution on the skeleton and bone health, and demonstrated that exposure to NOx represented as a major cause of skeletal damage, and showed a detrimental effect on LS-BMD [37]. A recent meta-analysis indicated that exposures to PM10, PM2.5, and NOx played negative roles in decreased BMD and increased the risk of osteoporotic fracture [38]. In addition, a retrospective cohort study has observed a positive association between ozone exposure and the risk of bone fracture development, potentially through ozone-induced oxidative stress injury that causes loss of bone mass [39]. This evidence suggests a positive association between long-term air pollutant exposure and bone damage, whereas, owing to the limitations of observational studies, they cannot rule out causality between air pollutants and bone health, thus previous observed findings may not always be generalizable.

In the current study, we conducted a two-phase MR study to investigate the causal effects of air pollutant exposure on the change in site-/age-specific BMD and the risk of bone fractures. We found that NOx exposures were causally associated with the decreased site-specific BMD of FN-BMD and TB-BMD; these findings were in line with some previous findings [38]. NOx is produced mainly from power plant emissions, vehicle exhausts, and truck exhausts. Once emitted into the atmosphere, NOx can undergo chemical reactions and enter the body through respiration. It has been proposed that NOx can disrupt the bone remodeling process by affecting oxidative stress, which contributes to cell dysfunction and potentially triggers inflammatory responses, leading to bone loss [40]. In addition, we found that exposure to PM10 has a detrimental effect on age-specific TB-BMD between 45 to 60 years, after adjusting for the confounding influences of PM2.5 and PM2.5–10; the findings supported the presence of direct causal associations between PM10 exposure and TB-BMD (45 < age < 60). As a matter of fact, the detrimental effect of PM10 exposure on bone health could be explained through several aspects. Evidence has shown that PM10 particles could not only induce chronic inflammation by enhancing the levels of pro-inflammatory cytokines but also generate oxidative stress, which is an imbalance between the production of free radicals and the body’s ability to counteract their harmful effects. Both chronic inflammation and oxidative stress can lead to impaired bone metabolism and cause bone damage [41]. Moreover, it has been shown that exposure to PM10 particles has been linked to hormonal disruption, as the crucial role of estrogen in the inhibition of osteoclastogenesis, any disruption of hormonal metabolism can negatively impact bone density and strength [42]. Notably, we recognized that our findings represented a higher effect size than results from studies examining the relationship between pollutants measured at temporal points and decreased BMD. This discrepancy might be attributed to the varied observed periods. In comparison to a previous study that showed a relatively short average period, our study used genetic variants to predict air pollutants that could reflect lifetime long-term exposure patterns, and the lifetime average period inherently incorporates the cumulative impact of exposure to air pollutants, providing a comprehensive perspective that aligns with the chronic nature of certain disease outcomes.

Air pollutants have been reported to induce oxidative stress and inflammation, which can further lead to disturbances in bone metabolism, reduction in bone density, and deterioration of bone structure [43,44]. It has been demonstrated that PM2.5 can be inhaled deep into the respiratory tract directly, and is transferred across the alveolar–capillary membrane, entering the bloodstream, triggering a local inflammatory response in the bone microenvironment, leading to imbalances in bone resorption and formation [45]. In addition, a prior study has shown that exposure to high concentrations of particulate matter enables the induction of the activations of various immune cells, including macrophages, natural killer (NK) cells and helper T cells, and leads to the release of several pro-inflammatory cytokines, such as tumor necrosis factor-alpha (TNF-α), interleukin-6 (IL-6), and monocyte chemoattractant protein-1 (MCP-1), suppressing the differentiation of osteoblast and inducing osteoclast differentiation, thereby increasing bone resorption and decreasing bone formation [46,47]. Furthermore, it has been indicated in the literature that air pollutant exposure, especially to particulate matter, can reduce sunlight penetration and, consequently, impact the synthesis of vitamin D, causing lower levels of vitamin D, which could disturb calcium absorption and metabolism and lead to impaired bone mineralization and density [48].

There are several limitations that need to be noted. First, both the datasets of exposure and outcome were derived from European ancestry, thus could give rise to concerns that our results may not be applicable to other populations, and further studies are necessary to validate the findings of our study in other ethnic populations. Second, due to the restriction of GWAS data availability, we were only able to use GWAS summary data on bone fractures; therefore, the association of air pollutants with the risk of site-specific bone fractures could not be well determined. Third, the study is limited by the fact that the measurement of air pollution exposure was only conducted in the ambient atmosphere, rather than obtaining more accurate levels of air pollutants directly in the circulatory system of humans, thus further restricting the in-depth exploration regarding the causal effects of individual biological exposure to air pollutants on bone health. Furthermore, due to the lack of detailed demographic information for BMD, the causal associations of air pollutants and gender-specific BMD/bone fracture are undetermined.

Despite the above limitations, our study also has its advantages. To the best of our knowledge, this is the first study that investigates causal associations between air pollutants exposure and BMD/bone fractures from a lifelong genetic perspective. Our study has a two-phase study design that implements both the univariate and multivariate MR analysis to assess the overall and direct causal effects of air pollutants on the phenotype of OP and bone fractures. In addition, the use of site-specific and age-specific BMD provides valuable insights into the specific impacts of air pollutants on BMD. By conducting site-specific BMD assessments, we could determine the localized effects of air pollutants on BMD in different sites. Additionally, age-specific BMD analysis allows for a better understanding of how air pollutants impact bone health across different age groups, and it could be helpful for identifying vulnerable populations and potential long-term effects.

## 5. Conclusions

This study elucidated the presence of causal adverse effects of NOx and PM10 exposure on decreased BMD, and found the directly causal impacts of single PM10 exposure on age-specific BMD with those aged 45 to 60 years. Our findings shed light on a better understanding of the relationship between air pollutants and bone health, providing additional evidence for the development of targeted interventions and strategies to mitigate negative impacts on bone health.

## Data Availability

The data and material that support the findings of this study are available from public datasets that could be found in IEU OPEN GWAS and Genetic Factors for Osteoporosis Consortium.

## Supplementary Materials

The following supporting information can be downloaded at: https://www.mdpi.com/article/10.3390/toxics12010027/s1, Supplementary Figure S1. The sample overlap between exposure and outcome datasets. PM: particulate matter; NO_X_: nitrogen oxides; BMD: bone mineral density; FA: forearm; FN: femoral neck; LS: lumbar spine; eBMD: estimated from quantitative heel ultrasounds BMD; TB: total body BMD. Supplementary Figure S2. The results of FDR correction for multiple tests. PM: particulate matter; NO_X_: nitrogen oxides; BMD: bone mineral density. Supplementary Figure S3. Sensitivity analyses for the causal effects of NOx on FN-BMD. NO_X_: nitrogen oxides; FN-BMD: femoral neck bone mineral density. Supplementary Figure S4. Sensitivity analyses for the causal effects of NOx on TB-BMD. NO_X_: nitrogen oxides; TB-BMD: total body bone mineral density. Supplementary Figure S5. Sensitivity analyses for the causal effects of PM10 on age-specific BMD (45 to 60 years). PM: particulate matter; BMD: bone mineral density. Supplementary Table S1. Single nucleotide polymorphisms (SNPs) associated with single air pollutants (used as IVs of Mendelian randomization). Supplementary Table S2. Causal effects of air pollutants on different site-specified BMD. Supplementary Table S3. Causal effects of air pollutants on different age-specified BMD. Supplementary Table S4. Causal effects of air pollutants on the risk of bone fractures.
