# Supplementary material for: Causal Relationships between Air Pollutant Exposure and Bone Mineral Density and the Risk of Bone Fractures: Evidence from a Two-Stage Mendelian Randomization Analysis
Source: Toxics. 2023 Dec 30;12(1):27. doi: 10.3390/toxics12010027 (PMC10820864; doi:10.3390/toxics12010027)
Supplement: Supplementary file 1 [file toxics-12-00027-s001.zip › toxics-2696690-Supplementary.pdf]

Supplementary Materials

Supplementary Table S1 SNPs associated with single air pollutants (used as IVs in MR analysis)

SNPs	Chr	Position	Effect allele	Other allele	EAF	Effect	SE	*P*-value	N	Phenotype	*F*-statistic	Variance explained (%)	Note
rs6749467	2	343517	A	G	0.466	-0.012	0.002	1.40E-08	423796	PM2.5	32.228	0.008	Removed from PM2.5/eBMD analysis
rs1372504	5	103749428	A	G	0.374	0.012	0.002	3.10E-08	423796	PM2.5	30.674	0.007	
rs12203592	6	396321	T	C	0.213	0.022	0.003	6.20E-17	423796	PM2.5	69.918	0.016	
rs77255816	6	20833602	T	C	0.037	0.031	0.006	4.20E-08	423796	PM2.5	30.041	0.007	Removed from PM2.5/FA-BMD and PM2.5/FN-BMD analysis
rs114708313	6	31329004	T	A	0.066	0.025	0.004	4.20E-08	423796	PM2.5	30.076	0.007	
rs77205736	8	10153460	T	C	0.274	0.014	0.002	2.10E-08	423796	PM2.5	31.399	0.007	
rs1537371	9	22099568	A	C	0.500	0.012	0.002	8.50E-09	423796	PM2.5	33.149	0.008	Removed from PM2.5/FA-BMD and PM2.5/FN-BMD analysis
rs72642437	18	45920421	T	C	0.004	0.113	0.019	3.10E-09	423796	PM2.5	35.119	0.008	
rs1748399	1	36655652	T	C	0.574	0.010	0.002	6.10E-06	423796	PM2.5-10	20.461	0.005	
rs76170056	1	3592598	A	C	0.111	0.017	0.003	1.90E-06	423796	PM2.5-10	22.736	0.005	
rs71323440	3	116072052	T	C	0.115	0.016	0.003	1.70E-06	423796	PM2.5-10	22.859	0.005	
rs9868243	3	113300863	C	T	0.195	0.012	0.003	5.70E-06	423796	PM2.5-10	20.587	0.005	
rs74561197	4	189419188	A	G	0.318	-0.010	0.002	6.80E-06	423796	PM2.5-10	20.253	0.005	
rs13125748	4	178069165	A	C	0.258	0.011	0.003	4.70E-06	423796	PM2.5-10	20.959	0.005	
rs9997134	4	3468969	C	T	0.399	-0.010	0.002	3.40E-06	423796	PM2.5-10	21.570	0.005	Removed from PM2.5-10/eBMD
rs138141967	4	110987693	T	G	0.015	-0.043	0.009	2.70E-06	423796	PM2.5-10	21.997	0.005	
rs116816317	4	133858337	A	G	0.020	0.038	0.008	2.30E-06	423796	PM2.5-10	22.342	0.005	
rs116259145	5	3279667	A	C	0.032	0.030	0.006	1.10E-06	423796	PM2.5-10	23.784	0.006	
rs72737245	5	32499805	G	A	0.233	-0.012	0.003	9.10E-06	423796	PM2.5-10	19.699	0.005	

rs78060907	5	37945741	A	C	0.020	-0.038	0.008	7.70E-07	423796	PM2.5-10	24.439	0.006	
rs62414841	6	63055986	T	C	0.029	0.029	0.007	7.60E-06	423796	PM2.5-10	20.025	0.005	
rs9497937	6	148423453	A	C	0.232	-0.013	0.003	7.20E-07	423796	PM2.5-10	24.555	0.006	
rs4716063	6	16381395	G	A	0.078	-0.019	0.004	5.80E-06	423796	PM2.5-10	20.543	0.005	
rs17675316	7	35316123	G	A	0.022	0.037	0.008	2.80E-06	423796	PM2.5-10	21.932	0.005	
rs111308789	7	13421408	A	T	0.057	-0.022	0.005	3.50E-06	423796	PM2.5-10	21.524	0.005	Removed from PM2.5-10/FA-BMD and PM2.5-10/FN-BMD analysis
rs1706918	7	105272307	A	G	0.099	0.018	0.004	6.00E-07	423796	PM2.5-10	24.906	0.006	
rs57048268	7	151623218	C	A	0.311	-0.011	0.002	3.20E-06	423796	PM2.5-10	21.705	0.005	
rs118101191	7	111672596	T	G	0.010	0.060	0.011	6.50E-08	423796	PM2.5-10	29.208	0.007	
rs1157546	8	18777006	C	T	0.053	-0.025	0.005	5.20E-07	423796	PM2.5-10	25.205	0.006	
rs10099257	8	132367285	T	C	0.230	-0.011	0.003	9.50E-06	423796	PM2.5-10	19.618	0.005	
rs7033430	9	93901159	G	T	0.764	-0.012	0.003	5.70E-06	423796	PM2.5-10	20.581	0.005	
rs117389221	9	133798921	C	T	0.017	-0.040	0.009	2.20E-06	423796	PM2.5-10	22.385	0.005	
rs7023300	9	16238128	A	T	0.339	-0.010	0.002	9.50E-06	423796	PM2.5-10	19.608	0.005	
rs79050669	12	78217999	T	G	0.034	0.027	0.006	7.10E-06	423796	PM2.5-10	20.178	0.005	Removed from PM2.5-10/FA-BMD and PM2.5-10/FN-BMD analysis
rs73220422	12	117175608	T	C	0.028	0.029	0.007	9.60E-06	423796	PM2.5-10	19.582	0.005	Removed from PM2.5-10/FA-BMD, PM2.5-10/eBMD and PM2.5-10/FN-BMD analysis
rs605027	12	72900322	T	C	0.730	-0.011	0.002	3.40E-06	423796	PM2.5-10	21.564	0.005	
rs143921782	13	39169251	C	T	0.010	0.050	0.011	9.00E-06	423796	PM2.5-10	19.707	0.005	
rs8006373	14	57123141	A	T	0.091	-0.018	0.004	3.30E-06	423796	PM2.5-10	21.659	0.005	
rs11621531	14	103577789	A	G	0.064	-0.023	0.004	2.50E-07	423796	PM2.5-10	26.565	0.006	
rs10152521	15	40386911	C	T	0.003	0.114	0.023	1.00E-06	423796	PM2.5-10	23.835	0.006	

rs74029037	15	92987146	T	C	0.037	0.026	0.006	8.00E-06	423796	PM2.5-10	19.940	0.005	Removed from PM2.5-10/FA-BMD, PM2.5-10/eBMD and PM2.5-10/FN-BMD analysis
rs8051340	16	11041413	G	C	0.123	0.016	0.003	1.30E-06	423796	PM2.5-10	23.356	0.006	Removed from PM2.5-10/FA-BMD, PM2.5-10/eBMD and PM2.5-10/FN-BMD analysis
rs62079137	17	76358269	C	T	0.107	-0.017	0.004	3.60E-06	423796	PM2.5-10	21.490	0.005	
rs9956831	18	74268809	T	C	0.014	-0.042	0.009	5.50E-06	423796	PM2.5-10	20.666	0.005	
rs12462492	19	22710669	T	G	0.257	-0.012	0.002	6.90E-07	423796	PM2.5-10	24.642	0.006	
rs117125329	19	53268990	G	C	0.008	0.055	0.012	4.20E-06	423796	PM2.5-10	21.162	0.005	Removed from PM2.5-10/FA-BMD and PM2.5-10/FN-BMD analysis
rs6078746	20	12737097	G	A	0.358	0.010	0.002	5.40E-06	423796	PM2.5-10	20.684	0.005	
rs6063430	20	48404077	C	T	0.331	-0.011	0.002	6.40E-06	423796	PM2.5-10	20.367	0.005	
rs4145410	22	22401006	G	A	0.838	-0.013	0.003	7.50E-06	423796	PM2.5-10	20.060	0.005	
rs114789974	2	19498078	A	C	0.010	-0.055	0.010	1.00E-08	455314	PM10	32.832	0.007	
rs182549	2	136616754	T	C	0.739	-0.012	0.002	2.10E-08	455314	PM10	31.357	0.007	Removed from PM10/FN-BMD analysis
rs13084230	3	164348898	T	C	0.200	-0.014	0.002	3.50E-08	455314	PM10	30.395	0.007	
rs6793835	3	135819934	A	G	0.264	-0.013	0.002	6.60E-09	455314	PM10	33.661	0.007	
rs56084453	3	44762830	G	A	0.210	0.015	0.002	5.90E-10	455314	PM10	38.352	0.008	Removed from PM10/FN-BMD analysis
rs4833095	4	38799710	C	T	0.207	0.025	0.002	1.70E-25	455314	PM10	108.964	0.024	Removed from PM10/FN-BMD and PM10/eBMD analysis
rs13122455	4	152891564	T	C	0.200	-0.014	0.002	1.30E-08	455314	PM10	32.293	0.007	
rs142169179	5	120443527	A	G	0.020	0.040	0.007	4.40E-08	455314	PM10	29.971	0.007	
rs62370429	5	101484521	C	T	0.058	-0.026	0.004	1.80E-09	455314	PM10	36.199	0.008	Removed from PM10/FA-BMD and PM10/FN-BMD analysis
rs2248162	6	29915061	C	T	0.640	0.012	0.002	7.80E-09	455314	PM10	33.331	0.007	
rs9640029	7	6685123	T	C	0.478	-0.014	0.002	2.70E-12	455314	PM10	48.898	0.011	

rs140295641	7	145875282	A	T	0.027	-0.035	0.006	1.30E-08	455314	PM10	32.406	0.007	
rs61620752	8	127488751	G	T	0.148	0.016	0.003	6.40E-09	455314	PM10	33.720	0.007	
rs2004679	8	10135193	C	T	0.308	0.012	0.002	2.50E-08	455314	PM10	31.049	0.007	
rs61875074	10	122622906	C	A	0.073	0.022	0.004	6.80E-09	455314	PM10	33.600	0.007	
rs147895162	11	83717507	C	T	0.015	-0.045	0.008	3.40E-08	455314	PM10	30.481	0.007	
rs10498638	14	93934435	C	T	0.188	0.014	0.003	3.30E-08	455314	PM10	30.513	0.007	
rs74247887	15	26213103	T	C	0.029	0.037	0.006	2.70E-10	455314	PM10	39.870	0.009	
rs74805019	15	53081859	C	G	0.034	-0.031	0.005	2.10E-08	455314	PM10	31.386	0.007	Removed from PM10/FA-BMD, PM10/eBMD and PM10/FN-BMD analysis
rs7200852	16	86157310	A	C	0.055	-0.024	0.004	4.00E-08	455314	PM10	30.129	0.007	
rs4788565	16	71892471	A	G	0.067	-0.022	0.004	4.10E-08	455314	PM10	30.111	0.007	
rs60304336	19	22794150	T	G	0.041	0.028	0.005	2.80E-08	455314	PM10	30.842	0.007	
rs7514956	1	74019696	C	A	0.187	-0.015	0.003	3.60E-08	456380	NOx	30.341	0.007	
rs6749467	2	343517	A	G	0.465	-0.012	0.002	2.60E-08	456380	NOx	30.958	0.007	
rs1318845	4	153001662	C	T	0.201	-0.014	0.003	4.80E-08	456380	NOx	29.802	0.007	
rs72808024	5	164479164	C	A	0.148	-0.017	0.003	4.60E-09	456380	NOx	34.336	0.008	
rs77255816	6	20833602	T	C	0.037	0.030	0.005	4.60E-08	456380	NOx	29.880	0.007	Removed from NOx/FA-BMD, NOx/eBMD and NOx/FN-BMD analysis
rs12203592	6	396321	T	C	0.219	0.019	0.002	3.20E-15	456380	NOx	62.164	0.014	
rs77205736	8	10153460	T	C	0.275	0.013	0.002	1.00E-08	456380	NOx	32.844	0.007	
rs1217106	8	64567670	G	A	0.782	0.015	0.003	7.50E-09	456380	NOx	33.405	0.007	

SNPs: single nucleotide polymorphisms; IVs: instrumental variables; Chr: chromosome; EAF: effect allele frequency; MR: mendelian randomization; SE: standard error of beta; PM: particulate matter; NO_X: nitrogen oxides; BMD: bone mineral density; FA: forearm; FN: femoral neck; LS: lumbar spine; eBMD: estimated heel BMD; TB: total body BMD;

Supplementary Table S2. Causal effects of air pollutants on site-specific BMD

Exposure	Outcome	SNPs (n)	Outcome sample size	IVW			Weighted Methods				MR egger regression				
							Weighted Median		Weighted Mode		MR egger		Intercept		
				Beta (SE)	*P* value	*P* heterogeneity	Beta (SE)	*P* value	Beta (SE)	*P* value	Beta (SE)	*P* value	Intercept	SE	*P* value
PM2.5	LS-BMD	8	44731 (GEFO)	0.07 (0.23)	0.764	0.592	0.17 (0.30)	0.567	0.20 (0.38)	0.623	0.197 (0.435)	0.667	-0.003	0.008	0.741
PM2.5	FA-BMD	6	10805 (GEFO)	0.21 (0.45)	0.638	0.991	0.35 (0.53)	0.506	0.39 (0.62)	0.550	0.550 (0.891)	0.565	-0.007	0.016	0.675
PM2.5	FN-BMD	6	49988 (GEFO)	-0.27 (0.26)	0.296	0.196	-0.00 (0.31)	0.750	0.01 (0.34)	0.980	0.325 (0.402)	0.465	-0.013	0.008	0.153
PM2.5	eBMD	7	456824 (GEFO)	-0.46 (0.32)	0.149	1.09E-34	-0.18 (0.10)	0.065	-0.09 (0.10)	0.390	-0.443 (0.844)	0.622	0.000	0.013	0.986
PM2.5	TB-BMD	8	66628 (GEFO)	-0.29 (0.13)	0.022	0.438	-0.23 (0.17)	0.187	-0.19 (0.18)	0.329	-0.193 (0.199)	0.370	-0.003	0.004	0.550
PM2.5-10	LS-BMD	41	44731 (GEFO)	0.07 (0.14)	0.154	0.971	0.09 (0.19)	0.636	0.12 (0.36)	0.745	-0.081 (0.317)	0.800	0.003	0.005	0.590
PM2.5-10	FA-BMD	35	10805 (GEFO)	0.14 (0.26)	0.805	0.801	0.06 (0.36)	0.875	-0.17 (0.76)	0.830	0.191 (0.632)	0.764	-0.001	0.009	0.929
PM2.5-10	FN-BMD	35	49988 (GEFO)	-0.06 (0.13)	0.661	0.913	-0.17 (0.17)	0.321	-0.30 (0.35)	0.398	-0.157 (0.290)	0.591	0.002	0.004	0.700
PM2.5-10	eBMD	37	456824 (GEFO)	-0.03 (0.03)	0.421	0.306	-0.07 (0.05)	0.126	-0.18 (0.10)	0.253	0.006 (0.071)	0.937	-0.001	0.001	0.629
PM2.5-10	TB-BMD	41	66628 (GEFO)	-0.03 (0.09)	0.728	0.290	0.10 (0.14)	0.489	0.08 (0.16)	0.615	0.027 (0.163)	0.870	-0.001	0.003	0.674
PM10	LS-BMD	22	44731 (GEFO)	-0.34 (0.16)	0.605	0.040	-0.27 (0.24)	0.244	-0.20 (0.35)	0.584	-0.01 (0.44)	0.991	-0.006	0.008	0.428
PM10	FA-BMD	20	10805 (GEFO)	-0.19 (0.31)	0.594	0.742	-0.36 (0.44)	0.411	-0.36 (0.67)	0.598	-0.74 (0.82)	0.375	0.011	0.015	0.475
PM10	FN-BMD	17	49988 (GEFO)	-0.29 (0.17)	0.088	0.987	-0.22 (0.22)	0.302	-0.16 (0.35)	0.655	-0.28 (0.43)	0.527	0.000	0.008	0.990

PM10	eBMD	20	456824 (GEFO)	-0.27 (0.14)	0.046	4.73E-45	-0.06 (0.06)	0.281	-0.039 (0.073)	0.602	0.391 (0.320)	0.238	-0.012	0.006	0.038
PM10	TB-BMD	22	66628 (GEFO)	-0.42 (0.12)	0.001	0.170	-0.28 (0.16)	0.079	-0.169 (0.254)	0.512	0.136 (0.313)	0.668	-0.011	0.006	0.071
NOx	LS-BMD	8	44731 (GEFO)	-0.38 (0.26)	0.040	0.485	0.01 (0.36)	0.971	0.061 (0.514)	0.908	-1.489 (1.201)	0.261	0.017	0.018	0.379
NOx	FA-BMD	7	10805 (GEFO)	-0.14 (0.57)	0.532	0.216	-0.24 (0.65)	0.711	-0.202 (0.922)	0.834	-0.387 (3.797)	0.923	0.004	0.056	0.950
NOx	FN-BMD	7	49988 (GEFO)	-0.71 (0.26)	0.006	0.312	-0.74 (0.33)	0.026	-0.185(0.627)	0.099	-1.218 (0.605)	0.091	-0.028	0.024	0.291
NOx	eBMD	7	456824 (GEFO)	-0.47 (0.31)	0.135	0.361	-0.24 (0.10)	0.013	-0.271 (0.149)	0.118	-0.255 (0.096)	0.875	-0.003	0.023	0.893
NOx	TB-BMD	8	66628 (GEFO)	-0.55 (0.18)	0.002	0.465	-0.67 (0.23)	0.004	-0.828 (0.407)	0.081	-0.829 (0.851)	0.367	0.004	0.013	0.752

SNPs: single nucleotide polymorphisms; IVW: inverse-variance weighted; SE: standard error; PM: particulate matter; NO_X: nitrogen oxides; BMD: bone mineral density; FA: forearm; FN: femoral neck; LS: lumbar spine; eBMD: estimated heel BMD; TB-BMD: total body BMD; GEFO: Genetic Factors for Osteoporosis Consortium.

Supplementary Table S3. Causal effects of air pollutants on age-specific BMD

Exposure	Outcome	SNPs (n)	Outcome sample size	IVW			Weighted Methods				MR egger regression				
							Weighted Median		Weighted Mode		MR egger		Intercept		
				Beta (SE)	*P* value	*P* heterogeneity	Beta (SE)	*P* value	Beta (SE)	*P* value	Beta (SE)	*P* value	Intercept	SE	*P* value
PM2.5	TB-BMD (age ≤ 15)	8	11807 (GEFO)	0.38（0.25）	0.524	0.089	0.56 (0.28）	0.049	0.79（0.40）	0.090	0.68 (0.66）	0.343	0.001	0.013	0.968
PM2.5	TB-BMD (15 < age ≤ 30)	8	4180 (GEFO)	0.42 (0.46)	0.367	0.455	0.50 (0.56)	0.377	0.50 (0.56)	0.404	0.46 (0.69)	0.527	-0.002	0.016	0.929
PM2.5	TB-BMD (30 < age ≤ 45)	8	9471 (GEFO)	-0.63 (0.30)	0.038	0.575	-0.70 (0.39)	0.069	-0.72 (0.39)	0.103	-0.79 (0.44)	0.124	0.005	0.010	0.633
PM2.5	TB-BMD (45 < age < 60)	8	18735 (GEFO)	-0.28 (0.25)	0.266	0.747	-0.34 (0.33)	0.311	-0.32 (0.37)	0.411	-0.14 (0.40)	0.739	-0.004	0.008	0.676
PM2.5	TB-BMD (age ≥ 60)	8	22435 (GEFO)	0.42 (0.46)	0.367	0.455	0.50 (0.56)	0.375	0.50 (0.57)	0.468	0.46 (0.69)	0.527	-0.002	0.016	0.929
PM2.5-10	TB-BMD (age ≤ 15)	41	11807 (GEFO)	-0.07 (0.18)	0.703	0.503	0.02 (0.31)	0.941	0.01 (0.28)	0.967	0.08 (0.30)	0.801	-0.003	0.005	0.554
PM2.5-10	TB-BMD (15 < age ≤ 30)	38	4180 (GEFO)	0.01 (0.33)	0.993	0.499	-0.08 (0.55)	0.882	-0.12 (0.53)	0.826	-0.08 (0.55)	0.892	0.002	0.010	0.862
PM2.5-10	TB-BMD (30 < age ≤ 45)	41	9471 (GEFO)	-0.20 (0.21)	0.350	0.969	0.07 (0.35)	0.836	0.01 (0.37)	0.983	-0.19 (0.37)	0.609	0.000	0.007	0.980
PM2.5-10	TB-BMD (45 < age < 60)	41	18735 (GEFO)	0.17 (0.19)	0.357	0.157	0.17 (0.27)	0.536	0.17 (0.44)	0.695	-0.05 (0.40)	0.905	0.004	0.006	0.534
PM2.5-10	TB-BMD (age ≥ 60)	38	22435 (GEFO)	0.01 (0.33)	0.993	0.499	-0.08 (0.56)	0.883	-0.12 (0.52)	0.820	-0.08 (0.55)	0.892	0.002	0.010	0.862
PM10	TB-BMD (age ≤ 15)	22	11807 (GEFO)	-0.07 (0.18)	0.901	0.503	0.02 (0.31)	0.941	0.01 (0.28)	0.967	0.08 (0.30)	0.801	-0.003	0.005	0.554
PM10	TB-BMD (15 < age ≤ 30)	22	4180 (GEFO)	0.01 (0.33)	0.643	0.499	-0.08 (0.55)	0.882	-0.12 (0.53)	0.826	-0.08 (0.55)	0.892	0.002	0.010	0.862
PM10	TB-BMD (30 < age ≤ 45)	22	9471 (GEFO)	-0.20 (0.21)	0.114	0.969	0.07 (0.35)	0.836	0.01 (0.37)	0.983	-0.19 (0.37)	0.609	0.000	0.007	0.980

PM10	TB-BMD (45 < age < 60)	22	18735 (GEFO)	-0.70 (0.21)	0.001	0.315	-0.84 (0.30)	0.005	-0.84 (0.40)	0.047	-0.56 (0.59)	0.350	-0.003	0.011	0.798
PM10	TB-BMD (age ≥ 60)	22	22435 (GEFO)	-0.21 (0.44)	0.643	0.376	-0.71 (0.63)	0.261	-0.71 (0.86)	0.417	0.46 (1.27)	0.723	-0.013	0.023	0.582
NOx	TB-BMD (age ≤ 15)	8	11807 (GEFO)	-0.88 (0.53)	0.096	0.943	-1.28 (0.55)	0.019	-1.55 (0.76)	0.083	-1.58 (2.76)	0.588	0.0107	0.0415	0.805
NOx	TB-BMD (15 < age ≤ 30)	8	4180 (GEFO)	0.37 (0.73)	0.614	0.610	0.81 (0.92)	0.379	0.92 (1.27)	0.491	1.71 (3.13)	0.624	-0.021	0.050	0.692
NOx	TB-BMD (30 < age ≤ 45)	8	9471 (GEFO)	-0.66 (0.47)	0.158	0.695	-0.56 (0.60)	0.338	-0.60 (0.94)	0.548	-3.13 (2.10)	0.186	0.038	0.032	0.272
NOx	TB-BMD (45 < age < 60)	8	18735 (GEFO)	-0.07 (0.43)	0.864	0.100	0.14 (0.46)	0.763	0.44 (0.75)	0.577	2.44 (1.82)	0.229	-0.039	0.028	0.207
NOx	TB-BMD (age ≥ 60)	8	22435 (GEFO)	0.37 (0.73)	0.614	0.610	0.81 (0.95)	0.395	0.92 (1.28)	0.495	1.71 (3.31)	0.624	-0.021	0.050	0.692

SNPs: single nucleotide polymorphisms; IVW: inverse-variance weighted; SE: standard error; PM: particulate matter; NO_X: nitrogen oxides; BMD: bone mineral density; FA: forearm; FN: femoral neck; LS: lumbar spine; eBMD: estimated heel BMD; TB-BMD: total body BMD; GEFO: Genetic Factors for Osteoporosis Consortium.

Supplementary Table S4. Causal effects of air pollutants on the risk of bone fractures

Exposure	Outcome	SNPs (n)	Outcome sample size	IVW			Weighted Methods				MR egger regression				
							Weighted Median		Weighted Mode		MR egger		Intercept		
				OR (95%CI)	*P* value	*P* heterogeneity	OR (95%CI)	*P* value	OR (95%CI)	*P* value	OR (95%CI)	*P* value	Intercept	SE	*P* value
PM2.5	Fractures	8	264973 (GEFO)	1.46 (0.90, 2.36)	0.126	0.079	1.75 (1.00, 3.05)	0.395	2.21 (1.00, 4.86)	0.495	1.98 (0.54, 7.29)	0.624	-0.006	0.011	0.632
PM2.5-10	Fractures	41	264973 (GEFO)	0.91 (0.74, 1.11)	0.143	0.432	0.98 (0.73, 1.30)	0.862	1.08 (0.57, 2.03)	0.822	0.54 (0.34, 0.84)	0.010	0.009	0.004	0.014
PM10	Fractures	22	264973 (GEFO)	1.19 (0.94, 1.50)	0.350	0.833	1.08 (0.78, 1.49)	0.641	0.95 (0.55, 1.66)	0.869	0.99 (0.55, 1.82)	0.999	0.003	0.006	0.549
NOx	Fractures	8	264973 (GEFO)	1.31 (0.82, 2.08)	0.257	0.139	1.29 (0.76, 2.18)	0.351	1.74 (0.69, 4.39)	0.278	1.14 (0.94, 2.85)	0.033	-0.032	0.013	0.047

SNPs: single nucleotide polymorphisms; IVW: inverse-variance weighted; OR: odds ratio; CI: confidence interval; SE: standard error; PM: particulate matter; NO_X: nitrogen oxides; BMD: bone mineral density; FA: forearm; FN: femoral neck; LS: lumbar spine; eBMD: estimated heel BMD; TB-BMD: total body BMD; GEFO: Genetic Factors for Osteoporosis Consortium.
