# Supplementary material for: Causal Relationships between Air Pollutant Exposure and Bone Mineral Density and the Risk of Bone Fractures: Evidence from a Two-Stage Mendelian Randomization Analysis
Source: Toxics. 2023 Dec 30;12(1):27. doi: 10.3390/toxics12010027 (PMC10820864; doi:10.3390/toxics12010027)
Supplement: Supplementary file 1 [file toxics-12-00027-s001.zip › toxics-2696690-Supplementary_STROBE-MR-checklist-fillable.pdf]

STROBE-MR checklist of recommended items to address in reports of Mendelian randomization studies[1 2]

Item No.	Section	Checklist item	Page No.	Relevant text from manuscript
1	**TITLE and ABSTRACT**	Indicate Mendelian randomization (MR) as the study's design in the title and/or the abstract if that is a main purpose of the study	2	This study was performed to systemically assess the genetically causal associations of air pollutants with site-/age-specific BMD and risk of bone fractures with the implementation of two-sample Mendelian randomization (TSMR) and multivariate Mendelian randomization (MVMR). The TSMR analysis was implemented to infer the causal associations between air pollutants and BMD and the risk of bone fractures, additional MVMR analysis was used to further estimate the direct causal effects of air pollutants on BMD, the occurrence of OP, and bone fractures.
	**INTRODUCTION**		4	
2	**Background**	Explain the scientific background and rationale for the reported study. What is the exposure? Is a potential causal relationship between exposure and outcome plausible? Justify why MR is a helpful method to address the study question	4	Osteoporosis (OP) is a chronic metabolic disease characterized by reduced bone mass, microarchitectural deterioration, and an increased risk of fragility fractures (Ensrud and Crandall, 2017). It poses a significant public health challenge, with approximately 200 million people suffering from OP each year (Xiao et al., 2022). Bone mineral density (BMD) measurements are recommended as the most reliable tool for the diagnosis of OP and assessment of bone health (Lupsa and Insogna, 2015). Age and gender-specific BMD measurements have suggested that both BMD and bone mass gradually decrease in the body with the ageing process, and showed a gender specificity in BMD decline trends (Grynpas, 1993). As a multifactorial disease, the exact etiology of OP is still not well understood; it has been revealed that genetic susceptibility, ageing, lifestyle, and medical conditions, etc., contribute to the onset and development of OP (Sfeir et al., 2022). In recent years, emerging evidence has shown that environmental factors may play an important role in the pathogenesis of OP (Wang et al., 2022).<br>Air pollutants, as important components of environmental factors, are defined as harmful concentrations of gaseous substances, particulate matter, and volatile substances (Shahrbaf et al., 2021). A large number of studies in the literature have demonstrated that short- or long-term exposure to air pollutants could cause chronic inflammation, induce the disturbance of oxidative stress and DNA damage, result in serious negative effects on human health, and lead to a series of disorders that are involved in the respiratory, cardiovascular, central nervous systems (Gumtorntip et al., 2023; Miller, 2020). A previous cohort study has revealed that exposure to air pollutants exhibited a harmful effect on

				bone health, where the high levels of air pollutant exposure were strongly associated with a reduced BMD, and increased risk of late-life bone fractures (Prada et al., 2017). A similar finding was also observed in a population-based retrospective cohort study, in which exposure to air pollutants increased the risk of the occurrence of OP from 39% to 89% in Taiwanese residents (Chang et al., 2015). Given the fact that several previous findings were based on observational studies, there remain, however, numerous unmeasured confounding factors and potential biases that might affect the validity and reliability of the observed associations, and the causal links between air pollution and BMD/bone fractures remain obscure.<br>Mendelian randomization (MR) is a cutting-edge statistical approach that leverages genetic variants as instrumental variables (IVs) to draw conclusions about causality between exposure and outcome. MR offers superior control of confounding factors and reverse causal associations compared to traditional observational studies, thus providing valuable genetic evidence for disease prevention and treatment (Bowden and Holmes, 2019). This innovative method holds promise for advancing our understanding of complex diseases and informing public health policies.
3	**Objectives**	State specific objectives clearly, including pre-specified causal hypotheses (if any). State that MR is a method that, under specific assumptions, intends to estimate causal effects	5	In the present study, we aimed to infer the causal associations between air pollution exposure and site-/age-specific BMD and the risk of bone fractures, with the implementation of two-sample MR (TSMR) and multivariate MR (MVMR) analyses, in order to identify and understand the causal roles of air pollution in the development of OP and bone fractures, which would be beneficial for improvements to prevention and the overall quality of life in these populations.
	**METHODS**			
4	**Study design and data sources**	Present key elements of the study design early in the article. Consider including a table listing sources of data for all phases of the study. For each data source contributing to the analysis, describe the following:	5-6	
	a)	Setting: Describe the study design and the underlying population, if possible. Describe the setting, locations, and relevant dates, including periods of recruitment, exposure, follow-up, and data collection, when available.	5-6	In MR analysis, single-nucleotide polymorphisms (SNPs) are commonly selected as IVs for estimates of exposure–outcome causal associations, but they must satisfy three key assumptions (Birney, 2022). First, the correlation assumption requires that IVs must be highly correlated with exposure factors in order to avoid the possible bias of weak IVs (Brion et al., 2013). Second, the exclusion restriction hypothesis must ensure that outcomes are solely influenced by exposure and not by any other factors, which means there is no potential for multiple causal pathways (Ding et al., 2017). The third, independence assumption requires that IVs

			should be free of confounding factors in the exposure–outcome association (Border et al., 2022).<br><br>The current study was a two-stage MR design (**Figure 1**), initially, the causal relationships of air pollutants with site- or age-specific BMD and the risk of bone fractures were evaluated using univariate MR analysis, where the exposure phenotype for air pollutants included particulate matter 2.5 (fine particulate matter less than 2.5 microns in diameter, PM2.5), particulate matter 2.5-10 (fine particulate matter between 2.5 and 10 microns in diameter, PM2.5-10), particulate matter 10 (fine particulate matter less than 10 microns in diameter, PM10) and nitrogen oxides (NOx). Given the observed causal associations between air pollutants and site- or age-specific BMD and the risk of bone fractures, further MVMR analysis was performed to explore the presence of the direct causal effects of single air pollutants, after adjusting for the confounding effect of other air pollutants.
b)	Participants: Give the eligibility criteria, and the sources and methods of selection of participants. Report the sample size, and whether any power or sample size calculations were carried out prior to the main analysis	6-7	Genetic instruments for air pollutants were obtained from the IEU OPEN GWAS database (https://gwas.mrcieu.ac.uk/), and we used IVs to explore the association between air pollutants and BMD, as well as fractures. The exposure datasets for three types of particulate matter (PM2.5, PM2.5-10, and PM10) were obtained from UK Biobank, which compiled 423,796 participants of European ancestry. The concentrations of PM2.5, PM10, and PM2.5-10 were estimated in use of land use regression (LUR) models, developed by the European Study of Cohorts for Air Pollution Effects (ESCAPE) project, at the home addresses of the participants (Eeftens et al., 2012). In addition, NOx data was obtained from the MRC-IEU database, which exported from the GWAS pipeline using UK Biobank's Pheasant-derived variables.
c)	Describe measurement, quality control and selection of genetic variants	7	Based on the three core assumptions of the MR analysis (correlation, independence, and exclusivity), we conditioned the selection of IVs to ensure the validity of causal estimates (Bowden et al., 2015). To be first, we only chose IVs from individuals of European ancestry to reduce potential bias from population stratification (Clarke et al., 2012). Subsequently, in accordance to the assumption of correlation, based on the threshold of $P < 5 \times 10^{8}$ and the linkage disequilibrium (LD) (r^2 = 0.01, kb = 10000), quantities of 8, 0, 22 and 8 genome-wide associated SNPs were selected for PM2.5, PM2.5-10, PM10 and NOx, respectively. However, as there was no available SNPs selected for PM2.5-10 at the level of $P < 5 \times 10^{8}$, we relaxed the threshold to $P < 1 \times 10^{-5}$ for IV selection and found 41 PM2.5-10 associated-SNPs. Due to the lack of available proxies in some of the exposure–outcome analyses, we excluded some SNPs as they were not present in the outcome

				**(Table1)**. In addition, the correlation strengths of enrolled IVs were assessed in using the *F* statistic, with the equation: $F = (R^2 \times (n-k-1)) / (k \times (1-R^2))$; $R^2 = 2 \times ((1 - MAF) \times MAF \times beta)$ (Burgess et al., 2011), and the results showed that the *F* value of each selected IVs was greater than 10, indicating that the selected IVs were not prone to the influence of weak IVs. Finally, there were a total of 79 air pollutants-associated SNPs, including 8 PM2.5-associated SNPs, 41 PM2.5-10-associated SNPs, 22 PM10-associated SNPs, 8 NOx-associated SNPs (**Table 1**).
		d) For each exposure, outcome, and other relevant variables, describe methods of assessment and diagnostic criteria for diseases	7-8	For the outcome datasets, we used BMD as outcome to represent the phenotype of OP, due to the varied BMD among the different body sites and age subgroups, the genetic summary data regarding five site-specific BMD measurements [lumbar spine BMD (LS-BMD), forearm BMD (FA-BMD), femoral neck BMD (FN-BMD), estimated from quantitative heel ultrasounds BMD (eBMD) and total body BMD (TB-BMD)] and five age-specific BMD measurements (age ≤ 15, 15 < age ≤ 30, 30 < age ≤ 45, 45 < age < 60 and age ≥ 60) were derived from the three large GWAS analysis consortiums based on reports by Zheng et al., Kemp et al., and Medina-Gomez et al., respectively (Birney, 2022; Liu et al., 2021; Locke et al., 2019), where the FN-BMD, LS-BMD, FA-BMD, TB-BMD, and five age-specific BMD measurements were measured by dual energy X-ray absorptiometry (DEXA), and eBMD was detected by quantitative ultrasonography. Considering the close relationships of OP and fractures, summary statistics for bone fractures were obtained from a publicly available GWAS of Morris et al. (Gregson et al., 2022). All of the genetic summary-level data were downloaded from Genetic Factors for Osteoporosis Consortium (GEFOS), and no additional ethical checks were therefore required.
		e) Provide details of ethics committee approval and participant informed consent, if relevant	8	This study was conducted based on the public open data from MRC-IEU database and the GEFO Osteoporosis Consortium, therefore no ethical review was required.
5	**Assumptions**	Explicitly state the three core IV assumptions for the main analysis (relevance, independence and exclusion restriction) as well assumptions for any additional or sensitivity analysis	7	Based on the three core assumptions of the MR analysis (correlation, independence, and exclusivity).

6	**Statistical methods: main analysis**	Describe statistical methods and statistics used	8-10	
		a) Describe how quantitative variables were handled in the analyses (i.e., scale, units, model)	9	Initially, the TSMR analysis was conducted to estimate the causal effects of four kinds of air pollutants (including PM2.5, PM10, PM2.5-10, and NOx) with site-/age-specific BMD and risk of bone fractures. The strength of genetic instruments for air pollutants correlations were quantified using the *F*-statistic, and the efficacy of the IVs was further assessed for all SNPs (Supplementary Table S1). Statistical power was computed with the implementation of online tool "mRnd" (https://shiny.cnsgenomics.com/mRnd/). The inverse variance weighted (IVW) and weighted median (WM) models were defined as the main analytical methods for the judgement of causal inference, and an additional MR-Egger model and weighted mode were also conducted. Among these four types of analytical methods, the IVW method presented as a reliable tool that could provide the precise causal effects between exposure and outcome, especially in the absence of horizontal pleiotropy (Burgess et al., 2015). In addition, considering the potential bias in the case of pleiotropy when performing IVW method, the WM method was constructed to verify the accuracy and stability of the results. When there were more than 50% invalid IVs present, the WM method yielded the most accurate results; it not only reduces the occurrence of Type I errors, but also provides a high degree of accuracy in assessing causal associations(Bowden et al., 2016). Considering the multiple tests during the causal inference between air pollutants and BMD/bone fractures, the false discovery rate (FDR) correction was implemented to adjust the *P*-values of the tests, in order to minimize the number of false positives (Shuken and McNerney, 2023). Both the IVW and WM results with $P < 0.05$ and FDR Q-value < 0.05 were defined as the presence of strong evidence of causality.
		b) Describe how genetic variants were handled in the analyses and, if applicable, how their weights were selected	9	Initially, the TSMR analysis was conducted to estimate the causal effects of four kinds of air pollutants (including PM2.5, PM10, PM2.5-10, and NOx) with site-/age-specific BMD and risk of bone fractures. The strength of genetic instruments for air pollutants correlations were quantified using the *F*-statistic, and the efficacy of the IVs was further assessed for all SNPs (Supplementary Table S1). Statistical power was computed with the implementation of online tool "mRnd" (https://shiny.cnsgenomics.com/mRnd/). The inverse variance weighted (IVW) and weighted median (WM) models were defined as the main analytical methods for the judgement of causal inference, and an

					additional MR-Egger model and weighted mode were also conducted. Among these four types of analytical methods, the IVW method presented as a reliable tool that could provide the precise causal effects between exposure and outcome, especially in the absence of horizontal pleiotropy (Burgess et al., 2015).
		c)	Describe the MR estimator (e.g., two-stage least squares, Wald ratio) and related statistics. Detail the included covariates and, in case of two-sample MR, whether the same covariate set was used for adjustment in the two samples	10	In addition, considering the potential bias in the case of pleiotropy when performing IVW method, the WM method was constructed to verify the accuracy and stability of the results. When there wre more than 50% invalid IVs present, the WM method yielded the most accurate results; it not only reduces the occurrence of Type I errors, but also provided a high degree of accuracy in assessing causal associations (Bowden et al., 2016). Considering the multiple tests during the causal inference between air pollutants and BMD/bone fractures, the false discovery rate (FDR) correction was implemented to adjust the *P*-values of the tests, in order to minimize the number of false positives (Shuken and McNerney, 2023). Both the IVW and WM results with $P < 0.05$ and FDR *Q*-value < 0.05 were defined as the presence of strong evidence of causality.
		d)	Explain how missing data were addressed	10	For IVs that were included in the exposure but not identified in the outcome, we harmonized after removing the corresponding IVs.
		e)	If applicable, indicate how multiple testing was addressed		
7	**Assessment of assumptions**		Describe any methods or prior knowledge used to assess the assumptions or justify their validity	10	In addition, considering the potential bias in the case of pleiotropy when performing IVW method, the WM method was constructed to verify the accuracy and stability of the results. When there were more than 50% invalid IVs present, the WM method yields the most accurate results; it not only reduced the occurrence of Type I errors, but also provided a high degree of accuracy in assessing causal associations (Bowden et al., 2016).
8	**Sensitivity analyses and additional analyses**		Describe any sensitivity analyses or additional analyses performed (e.g., comparison of effect estimates from different approaches, independent replication, bias analytic techniques, validation of instruments, simulations)	10	To ensure the stability and reliability of our results, we conducted sensitivity analyses using several methods. First, to investigate possible horizontal pleiotropy, the MR Pleiotropy RESidual Sum and Outlier (MR-PRESSO) method was employed to detect and correct for horizontal pleiotropy (Ding et al., 2017), and it was found that there was no significantly horizontal pleiotropy with the *P*-value greater than 0.05, indicating that IVs did not affect the outcome through pathways independent of exposure (Pereira et al., 2010). Second, Cochran's *Q* statistic was calculated to assess the degree of heterogeneity among the included IVs, where a *P*-value > 0.05 indicated no marked heterogeneity

					among the IVs, suggesting that the causal associations were not influenced by the individual SNP effects (Pereira et al., 2010). In addition, the leave-one-out (LOO) method was used to further evaluate the robustness of causality by excluding single SNPs at the time of the analysis and reassessing the impact on the overall causal estimates (Hemani et al., 2018).
9	**Software and pre-registration**			11	
		a)	Name statistical software and package(s), including version and settings used	11	All statistical analyses were conducted using R version 4.2.2 software with the implementation of the "TwoSampleMR", "MRPRESSO", and "MendelianRandomization" packages. All results were visualized in the form of scatter plots, forest plots, funnel plots, and leave-one-out plots, with the use of the "ggplot2" and "forestplot" packages.
		b)	State whether the study protocol and details were pre-registered (as well as when and where)		Pre-registration is not yet available.
	**RESULTS**			11-12	
10	**Descriptive data**				
		a)	Report the numbers of individuals at each stage of included studies and reasons for exclusion. Consider use of a flow diagram	11	Given the aforementioned criteria of IV selection, 79 air pollutant-associated SNPs were screened for causal estimates in the present study, which comprised of 8 PM2.5-associated SNPs, 41 PM2.5-10-associated SNPs, 22 PM10-associated SNPs, and 8 NOx-associated SNPs, respectively (Table 1).
		b)	Report summary statistics for phenotypic exposure(s), outcome(s), and other relevant variables (e.g., means, SDs, proportions)	11	The *F*-statistics for PM2.5, PM2.5-10, PM10, and NOx were 34.526, 36.534, 21.925, and 35.466, respectively, suggesting that the selected IVs were sufficiently robust, and were not prone to the influence of weak IVs.
		c)	If the data sources include meta-analyses of previous studies, provide the assessments of heterogeneity across these studies		
		d)	For two-sample MR:	11	Moreover, the calculation of participant overlap found a lower sample overlap between the datasets of air pollutants and OP/bone fractures,

			i. Provide justification of the similarity of the genetic variant-exposure associations between the exposure and outcome samples<br>ii. Provide information on the number of individuals who overlap between the exposure and outcome studies		indicating that the causal estimates of the present study were less likely to be affected by Winner's curse bias.
11	**Main results**				
		a)	Report the associations between genetic variant and exposure, and between genetic variant and outcome, preferably on an interpretable scale		
		b)	Report MR estimates of the relationship between exposure and outcome, and the measures of uncertainty from the MR analysis, on an interpretable scale, such as odds ratio or relative risk per SD difference	11	The results of the IVW method together with the FDR correction revealed that NOx exposures were causally linked with the lower FN-BMD (Beta = -0.71, 95%CI: -1.22, -0.20, $P = 0.006$) and TB-BMD (Beta = -0.55, 95%CI: -0.90, -0.21, $P = 0.002$) (Figure 2 & Supplementary Figure 2), the above findings were also supported by the WM method (Supplementary Table 1). In addition, we did not detect the presence of horizontal pleiotropy and marker heterogeneity (both $P > 0.05$) (Supplementary Table 1). The sensitivity analysis with the LOO method revealed that no single SNPs drove these results after stepwise elimination of individual SNPs (Supplementary Figure 3 & 4).<br>In order to further investigate the causal effects of air pollutants on age-specific BMD, five subgroups of age-specific BMD (including age ≤ 15, 15 < age ≤ 30, 30 < age ≤ 45, 45 < age < 60 and age ≥ 60) were used to represent the phenotype outcome for causal inference. The results of the IVW method and FDR correction observed a negatively causal association of PM10 exposure with decreased BMD in the 45 to 60 years age group (Beta = -0.70, 95%CI: -1.12, -0.29, $P = 0.001$) (Figure 3 & Supplementary Figure S5), further WM method analysis supported the robustness of the IVW results (Supplementary Table 2).<br>As previously described, OP patients were at a higher risk of the occurrence of bone fractures; therefore, the additional TSMR analysis was performed to infer the causality between air pollutants and risk of bone fractures, and the results indicated that there were no obviously causal effects of air pollutant exposure on the risk of bone fractures (Figure 4 & Supplementary Table 3).
		c)	If relevant, consider translating estimates of relative risk into absolute risk for a meaningful time period	12	Considering the possibility of interactional effects of multiple particulate matters on BMD simultaneously, the MVMR analysis was implemented to evaluate the direct effects of PM10 on age-specific BMD (45< age < 60) after correcting for the influence of PM2.5 and PM2.5-10. The results of the MVMR analysis found that single exposure to PM10 had a directly causal effect on decreased BMD in the subgroup aged between 45 to 60 years (Beta = -0.91, 95%CI: -1.30, -

				0.51, $P = 4.05 \times 10^{-5}$) (Figure 5), suggesting that there was an independent effect of PM10 exposure on bone damage in the vulnerable population aged between 45 to 60 years.
		d) Consider plots to visualize results (e.g., forest plot, scatterplot of associations between genetic variants and outcome versus between genetic variants and exposure)	12	Figure 3 & Supplementary Figure S5<br>Figure 4 & Supplementary Table 3<br>Supplementary Table 2
12	**Assessment of assumptions**			
		a) Report the assessment of the validity of the assumptions	11	The *F*-statistics for PM2.5, PM2.5-10, PM10, and NOx were 34.526, 36.534, 21.925, and 35.466, respectively, suggesting that the selected IVs were sufficiently robust and were not prone to the influence of weak IVs.
		b) Report any additional statistics (e.g., assessments of heterogeneity across genetic variants, such as I^2, Q statistic or E-value)	12	In addition, we did not detect the presence of horizontal pleiotropy and marker heterogeneity (both $P > 0.05$). The sensitivity analysis using the LOO method revealed that no single SNPs drove these results after stepwise elimination of individual SNPs.
13	**Sensitivity analyses and additional analyses**			
		a) Report any sensitivity analyses to assess the robustness of the main results to violations of the assumptions	12	In addition, we did not detect the presence of horizontal pleiotropy and marker heterogeneity (both $P > 0.05$).
		b) Report results from other sensitivity analyses or additional analyses	12	The sensitivity analysis using the LOO method revealed that no single SNPs drove these results after stepwise elimination of individual SNPs.
		c) Report any assessment of direction of causal relationship (e.g., bidirectional MR)		
		d) When relevant, report and compare with estimates from non-MR analyses		
		e) Consider additional plots to visualize results (e.g., leave-one-out analyses)		Supplementary Figure 3 & 4
	**DISCUSSION**		13-15	
14	**Key results**	Summarize key results with reference to study objectives	14	In the current study, we conducted a two-phase MR study to investigate the causal effects of air pollutants exposure on the change of site-/age-specific BMD and the risk of bone fractures. We found that NOx

				exposures were causally associated with the decreased site-specific BMD of FN-BMD and TB-BMD; these findings were in line with some previous findings (Mousavibaygei et al., 2023). NOx is produced mainly from power plant emissions, vehicle exhausts and truck exhausts. Once emitted into the atmosphere, NOx can undergo chemical reactions and enter the body through respiration. It has been proposed that NOx can disrupt bone remodeling process by affecting oxidative stress, which contributed to cell dysfunction and potentially triggers inflammatory responses, leading to bone loss (Adami et al., 2022). In addition, we found that exposure to PM10 has a detrimental effect on age-specific BMD between 45 to 60 years, after adjusting for the confounding influence of PM2.5 and PM2.5-10; the findings supported the presence of directly causal associations between PM10 exposure and BMD (45< age <60). As a matter of fact, the detrimental effect of PM10 exposure on bone health could be explained through several aspects. Evidence has shown that PM10 particles could not only induce the chronic inflammation by enhancing the levels of pro-inflammatory molecules, but also generate the oxidative stress, which is an imbalance between the production of free radicals and the body's ability to counteract their harmful effects. Both the chronic inflammation and oxidative stress can lead to an impaired bone metabolism and cause bone damage (Briot et al., 2017). Moreover, it has been shown that exposure to PM10 particles has been linked to hormonal disruption, as the crucial role of estrogen in the inhibition of osteoclastogenesis, any disruption of hormonal metabolism can negatively impact bone density and strength (Almeida et al., 2017).
15	**Limitations**	Discuss limitations of the study, taking into account the validity of the IV assumptions, other sources of potential bias, and imprecision. Discuss both direction and magnitude of any potential bias and any efforts to address them	15	There are several limitations that need to be noted. First, both the datasets of exposure and outcome were derived from the European ancestry, thus it could give rise to concerns that our results may not be applicable to other populations, and further studies are necessary to validate the findings of our study in other ethnic populations. Second, due to the restriction of GWAS data availability, we were only able to use GWAS summary data on bone fractures; therefore, the association of air pollutants with the risk of site-specific bone fractures could not be well determined. Third, the study is limited by the fact that the measurement of air pollution exposure was only conducted in the ambient atmosphere, rather than obtaining more accurate levels of air pollutants directly in the circulatory system of humans, thus further restricted the in-depth exploration regarding the causal effects of individual biological exposure to air pollutants on bone health.
16	**Interpretation**			

a)	Meaning: Give a cautious overall interpretation of results in the context of their limitations and in comparison with other studies	16	Despite the above limitations, our study also has its advantages. To the best of our knowledge, this is the first study that investigate causal association between air pollutants exposure and BMD/bone fractures from a lifelong genetic perspective. Our study has a two-phase study design that implements both the univariate and multivariate MR analysis to assess the overall and direct causal effects of air pollutants on the phenotype of OP and bone fractures. In addition, the use of site-specific and age-specific BMD provides valuable insights into the specific impacts of air pollutants on BMD. By conducting site-specific BMD assessments, we can determine the localized effects of air pollutants on BMD in different sites. Additionally, age-specific BMD analysis allows for a better understanding of how air pollutants impact bone health across different age groups, and it could be helpful for identifying vulnerable populations and potential long-term effects.
b)	Mechanism: Discuss underlying biological mechanisms that could drive a potential causal relationship between the investigated exposure and the outcome, and whether the gene-environment equivalence assumption is reasonable. Use causal language carefully, clarifying that IV estimates may provide causal effects only under certain assumptions	13	Previous studies have shown that long-term exposure to air pollution can have negative effects on bone health. Specifically, it has been found that exposure to particulate matter and nitrogen dioxide (NO2), two common components of air pollutants, can lead to reduced BMD and increased risk of bone fractures in later life (Alvaer et al., 2007). Prada et al. firstly explored the effects of air pollution on the skeleton and bone health, and demonstrated that exposure to NOx represented as a major cause of skeletal damage, and showed a detrimental effect on LS-BMD (Prada et al., 2023). A recent meta-analysis indicated that exposures to PM10, PM2.5 and NOx played negative roles in decreased BMD and increased the risk of osteoporotic fracture (Mousavibaygei et al., 2023). In addition, a retrospective cohort study has observed a positive association between ozone exposure and the risk of bone fractures development, potentially through the ozone-induced oxidative stress injury that causes the loss of bone mass (Lu et al., 2023). This evidence suggests a positive association between long-term air pollutants exposure and bone damage, whereas, owing to the limitations of observational studies, they cannot rule out causality between air pollutants and bone health, thus previously observed findings may not always be generalizable.
c)	Clinical relevance: Discuss whether the results have clinical or public policy relevance, and to what extent they inform effect sizes of possible interventions	15	Our study has a two-phase study design that implements both univariate and multivariate MR analysis to assess the overall and direct causal effects of air pollutants on the phenotype of OP and bone fractures. In addition, the use of site-specific and age-specific BMD provides valuable insights into the specific impacts of air pollutants on BMD. By conducting site-specific BMD assessments, we can determine the localized effects of air pollutants on BMD in different

				sites. Additionally, age-specific BMD analysis allows for a better understanding of how air pollutants impact bone health across different age groups, and it could be helpful for identifying vulnerable populations and potential long-term effects.
17	**Generalizability**	Discuss the generalizability of the study results (a) to other populations, (b) across other exposure periods/timings, and (c) across other levels of exposure		
	**OTHER INFORMATION**			
18	**Funding**	Describe sources of funding and the role of funders in the present study and, if applicable, sources of funding for the databases and original study or studies on which the present study is based	16	This study was funded by grants from the Key Scientific Research Foundation of the Education Department of the Province Anhui (2022AH050653) and the Natural Science Foundation of Anhui Medical University (2022xkj006).
19	**Data and data sharing**	Provide the data used to perform all analyses or report where and how the data can be accessed, and reference these sources in the article. Provide the statistical code needed to reproduce the results in the article, or report whether the code is publicly accessible and if so, where	17	The data and material that support the findings of this study are available from public datasets that can be found in IEU OPEN GWAS and the Genetic Factors for Osteoporosis Consortium.
20	**Conflicts of Interest**	All authors should declare all potential conflicts of interest	16	Xiao Hu, Yan Zhao, Tian He, Zhao-Xing Gao, Peng Zhang, Yang Fang, Man Ge, Yi-Qing Xu, Hai-Feng Pan, Peng Wang declare that they have no relevant affiliations or financial involvement with any organization or entity with a financial interest in or financial conflicts with the subject matter or materials discussed in the manuscript.

This checklist is copyrighted by the Equator Network under the Creative Commons Attribution 3.0 Unported (CC BY 3.0) license.

1. Skrivankova VW, Richmond RC, Woolf BAR, Yarmolinsky J, Davies NM, Swanson SA et al. Strengthening the Reporting of Observational Studies in Epidemiology using Mendelian Randomization (STROBE-MR) Statement. JAMA. 2021;under review.

2. Skrivankova VW, Richmond RC, Woolf BAR, Davies NM, Swanson SA, VanderWeele TJ et al. Strengthening the Reporting of Observational Studies in Epidemiology using Mendelian Randomisation (STROBE-MR): Explanation and Elaboration. BMJ. 2021;375:n2233.
