# Supplementary figures and images for: Causal Relationships between Air Pollutant Exposure and Bone Mineral Density and the Risk of Bone Fractures: Evidence from a Two-Stage Mendelian Randomization Analysis
Source: Toxics. 2023 Dec 30;12(1):27. doi: 10.3390/toxics12010027 (PMC10820864; doi:10.3390/toxics12010027)

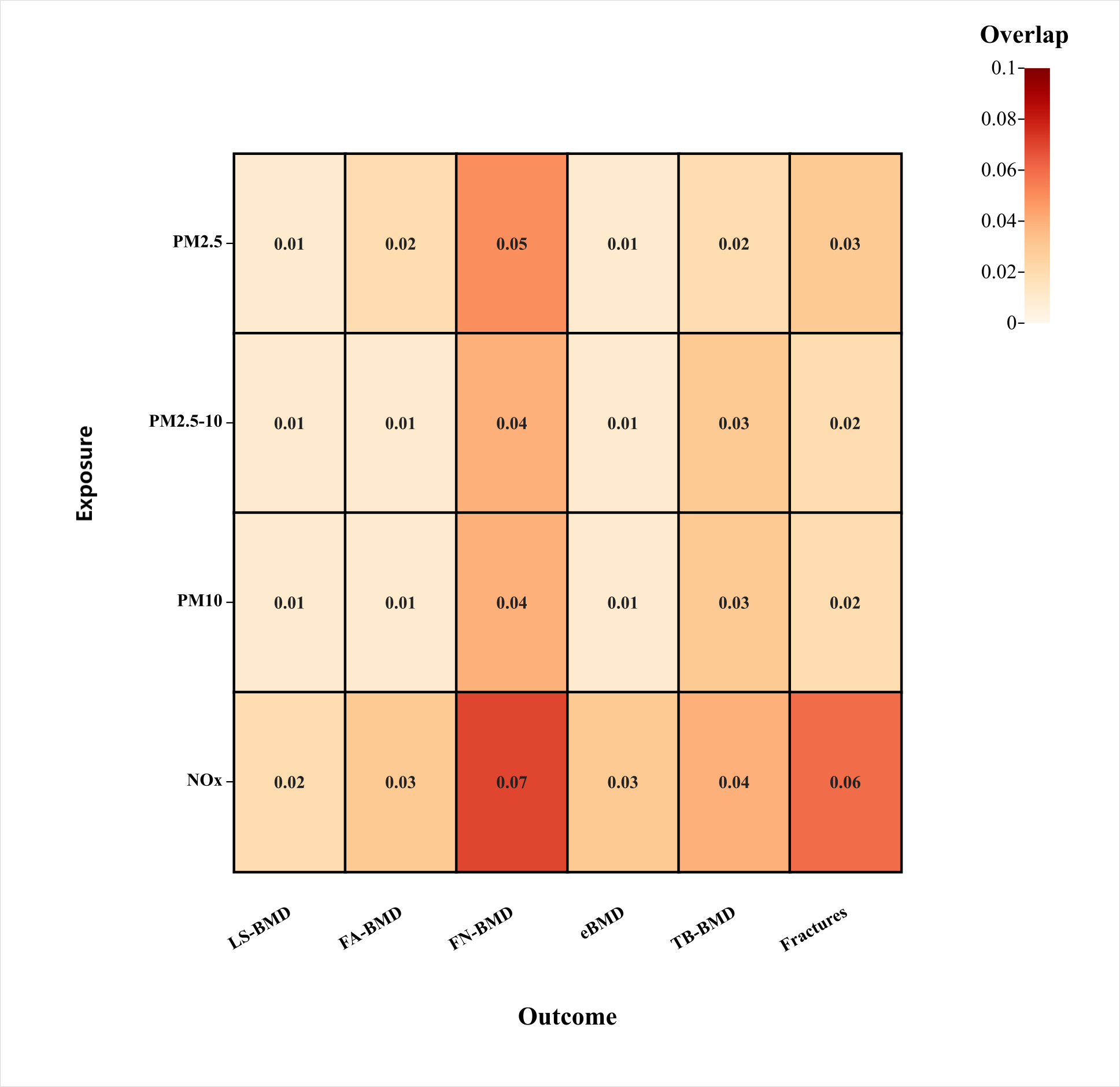

Supplement: Supplementary file 1 [file toxics-12-00027-s001.zip › Supplementary Figure S1.tiff]

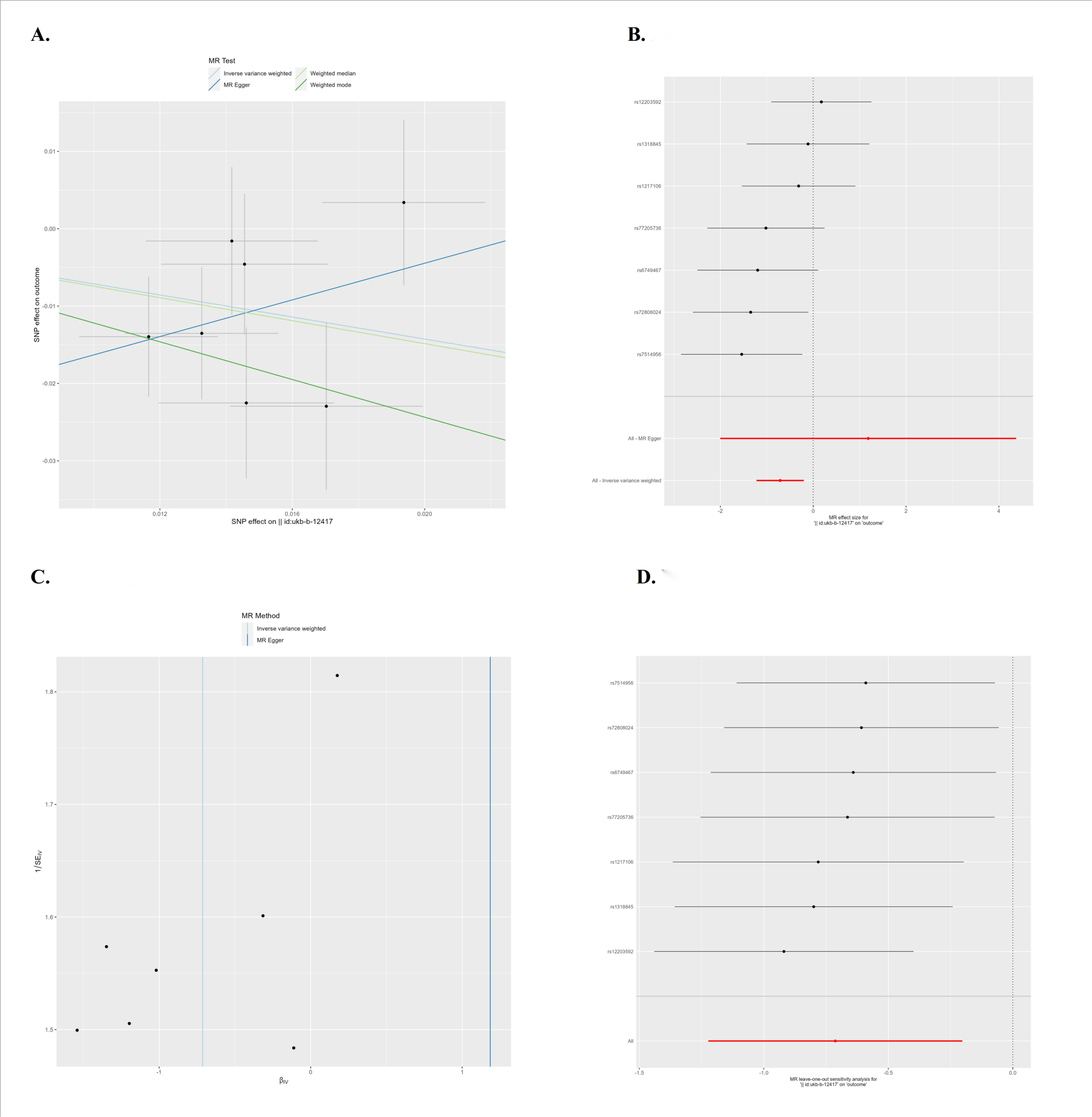

Supplement: Supplementary file 1 [file toxics-12-00027-s001.zip › Supplementary Figure S3.tiff]

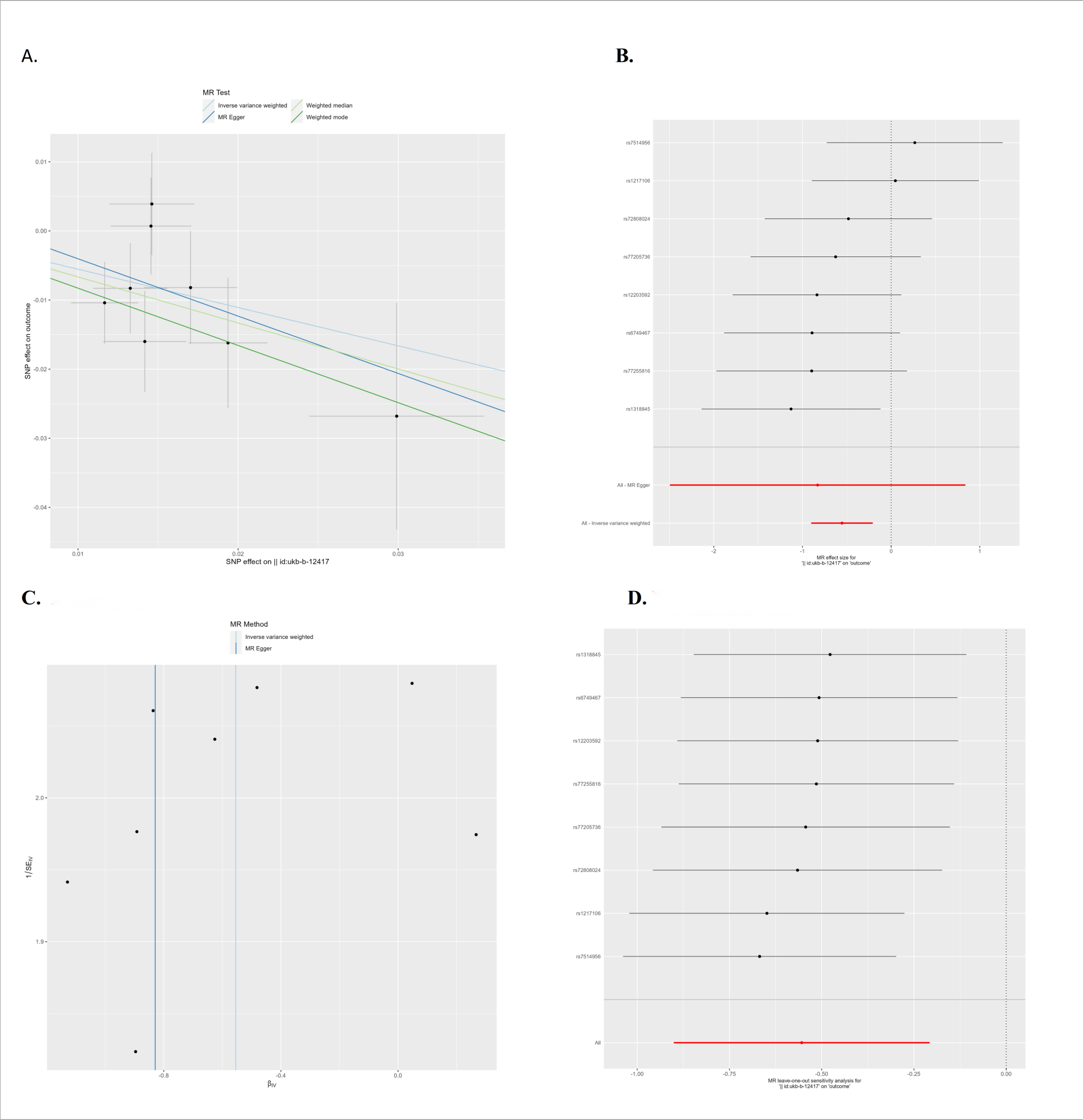

Supplement: Supplementary file 1 [file toxics-12-00027-s001.zip › Supplementary Figure S4.tiff]

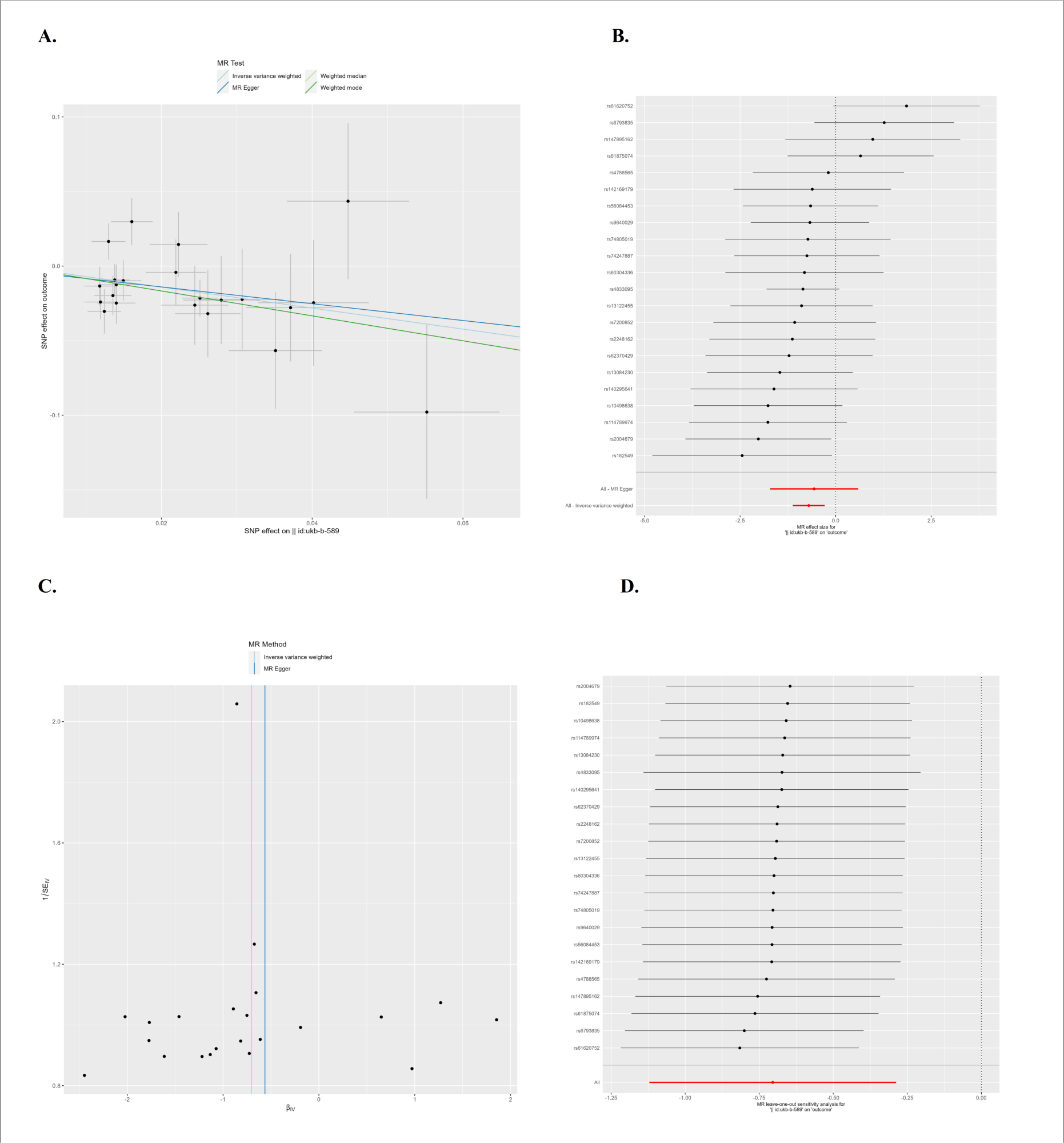

Supplement: Supplementary file 1 [file toxics-12-00027-s001.zip › Supplementary Figure S5.tiff]
